# Using the Knowledge of Post-transcriptional Regulations to Guide Gene Selections for Molecular Breeding in Soybean

**DOI:** 10.3389/fpls.2022.867731

**Published:** 2022-03-31

**Authors:** Yee-Shan Ku, Ming-Yan Cheung, Sau-Shan Cheng, Muhammad Azhar Nadeem, Gyuhwa Chung, Hon-Ming Lam

**Affiliations:** ^1^Centre for Soybean Research of the State Key Laboratory of Agrobiotechnology and School of Life Sciences, The Chinese University of Hong Kong, Shatin, Hong Kong SAR, China; ^2^Faculty of Agricultural Sciences and Technologies, Sivas University of Science and Technology, Sivas, Turkey; ^3^Department of Biotechnology, Chonnam National University, Yeosu, South Korea

**Keywords:** post-transcriptional gene regulation, non-coding RNA, translational regulation, protein modification, soybean, molecular breeding

## Abstract

The omics approaches allow the scientific community to successfully identify genomic regions associated with traits of interest for marker-assisted breeding. Agronomic traits such as seed color, yield, growth habit, and stress tolerance have been the targets for soybean molecular breeding. Genes governing these traits often undergo post-transcriptional modifications, which should be taken into consideration when choosing elite genes for molecular breeding. Post-transcriptional regulations of genes include transcript regulations, protein modifications, and even the regulation of the translational machinery. Transcript regulations involve elements such as microRNAs (miRNAs) and long non-coding RNAs (lncRNAs) for the maintenance of transcript stability or regulation of translation efficiency. Protein modifications involve molecular modifications of target proteins and the alterations of their interacting partners. Regulations of the translational machinery include those on translation factors and the ribosomal protein complex. Post-transcriptional regulations usually involve a set of genes instead of a single gene. Such a property may facilitate molecular breeding. In this review, we will discuss the post-transcriptional modifications of genes related to favorable agronomic traits such as stress tolerance, growth, and nutrient uptake, using examples from soybean as well as other crops. The examples from other crops may guide the selection of genes for marker-assisted breeding in soybean.

## Introduction

Soybean seed is a food crop which is uniquely rich in protein, oil, and isoflavones (Hassan, 2013). Soybean germplasm collections featuring desirable traits in terms of seed quality and yield have been curated (Qiu et al., 2013). Seed quality-related traits include the absence of allergens, desirable seed isoflavone contents, and desirable 11S/7S globulin ratios, while yield-related traits include appropriate plant architecture and seed size for cultivation and harvesting (Qiu et al., 2013). Hence, gene discovery and allele mining have been focused on traits such as yield, protein content, oil content, as well as salt tolerance, drought tolerance, low-temperature tolerance, soybean cyst nematode resistance, and soybean mosaic virus resistance (Qiu et al., 2013). Soybean germplasms featuring different desirable traits have been suggested to be the reservoir for breeding. Recently, traits including appropriate plant height for lodging resistance, short internode length, greater number of internodes, lower number of branches, moderate pod numbers, high podding rate, higher ratio of pods with four seeds, moderate 100-seed weight, small petiole angle, and short petiole length are also included as favorable traits for soybean breeding in the “soybean green revolution” (Liu et al., 2020a).

“Pyramiding,” which refers to the assembly of multiple desired genes into a single genotype, has long been employed as a molecular breeding strategy (Dormatey et al., 2020). Recently, based on genome sequences, a new breeding strategy named “Potalaization” has been adopted, in which elite cultivars with high agronomic performance as well as genetic diversity were selected as candidate parental materials for soybean breeding (Qi et al., 2021). Molecular breeding refers to the breeding by genetic manipulation at the DNA level (Moose and Mumm, 2008; Jiang, 2013). The strategies include marker-assisted selection and crossing and could also include genetic modifications (Moose and Mumm, 2008; Jiang, 2013). Genomic and transcriptomic data have provided valuable information for the design of molecular breeding strategies. Nevertheless, there is increasing evidence suggesting the regulation of important agronomic traits at the post-transcriptional level. In this review, examples of post-transcriptional regulations, including transcript regulation, translational regulation, and post-translational modification, will be discussed in the context of stress tolerance and nutrient uptake. Post-translational modifications usually result in quick alterations of protein functions or involve the regulation of a broad set of genes by a single regulator. Such properties may be beneficial for molecular breeding, which usually aims to stack several favorable traits in the same cultivar. Hence, genes involved in this type of post-translational regulations are sought-after candidates for the molecular breeding of soybean.

## Non-coding RNAs Are Regulators of Stress Responses in Soybean

As previously reviewed, non-coding RNAs (ncRNAs) have been recognized as transcription regulators (Zhang et al., 2019). According to their lengths, ncRNAs are classified as either long non-coding RNAs (lncRNAs) which are longer than 200 nt or small non-coding RNAs (sncRNAs) which are shorter than 200 nt. sncRNAs are further classified as microRNAs (miRNAs), small interfering RNAs (siRNAs), or piwi-interacting RNAs (piRNAs). ncRNAs can interact with mRNAs to promote or repress mRNA expression, or interact with other ncRNAs as a sponge to regulate the ncRNA functions. ncRNAs can also interact with each other to regulate the cleavage of coding transcripts. For example, lncRNAs have been shown to interact with miRNAs which mediate transcript cleavage (Lu et al., 2020). lncRNAs have also been reported to direct DNA methylation in plant (Kong et al., 2020; de Oliveira Urquiaga et al., 2021).

A study that integrated the annotations and analyses of small RNA-producing loci from 47 plant species found that the siRNA22 loci occupy a considerable proportion of the genomes of species in the asterids clade (Lunardon et al., 2020). Unexpectedly, although soybean is not in the asterids clade, it also has abundant siRNA22 loci in its genome (Lunardon et al., 2020). Such a property opens the door to siRNA manipulation in soybean molecular breeding for desired traits. In soybean, studies have shown that ncRNAs, including lncRNAs, miRNAs, and siRNAs, modulate gene expressions by regulating transcript levels in response to stresses, growth, and nutrient acquisition of the plant roots (Pan et al., 2016; Sun et al., 2019, 2020; Zhou et al., 2020; Fan et al., 2021; Lei et al., 2021; Niu et al., 2021). Examples will be discussed below.

### The Expressions of ncRNAs Are Responsive to Stresses

Stress-responsive ncRNAs have been identified in various plant species. Recently, a database curating experimentally validated stress-responsive ncRNAs in plants was constructed (Wu et al., 2020). The database contains 4,227 entries of ncRNAs, including miNRAs, lncRNAs, and circular RNAs, covering 48 biotic and 91 abiotic stress conditions (Wu et al., 2020). In soybean, lncRNAs responsive to stresses, including drought, salt, alkaline, and CO_2_ concentration, have been reported (Lin et al., 2020). Soybean lncRNAs were also found to have germplasm-specific stress responses (Lin et al., 2020). Although the potential target transcripts regulated by these lncRNAs remain unclear, the lncRNA datasets from different soybean germplasms under different conditions will provide a reference for studies on soybean transcript regulation. Soybean miRNAs responsive to common biotic interactors such as rhizobia (Yan et al., 2016; Fan et al., 2021) and cyst nematode (Li et al., 2012; Tian et al., 2017) have also been reported. The observation that soybean ncRNAs are responsive to stresses suggests their involvement in these stress responses.

### The Regulation of the Adaptation to Stresses by ncRNAs in Soybean

#### Abiotic Stress

##### lncRNA

In soybean, lncRNA77580 was found to be localized in the nucleus, and its expression level was repressed in roots after NaCl treatment (Niu et al., 2021). The overexpression or large-fragment deletion of lncRNA77580 led to the altered expressions of its neighboring genes, including genes encoding antimicrobial peptides and receptor-like protein kinases, in the transgenic hairy roots (Niu et al., 2021). Based on this pattern of regulation, when designing a strategy for molecular breeding, the whole segment of the genome, including the lncRNA and its neighboring regulated genes, has to be taken into consideration.

##### miRNA

Compared to lncRNAs, the regulatory functions of miRNAs in soybean are more well-known. In a degradome-seq analysis of soybean treated with PEG-simulated drought, 1,000 transcripts from 384 genes targeted by 365 miRNAs were found (Zhou et al., 2020). Among these miRNAs, gma-MIR398c was found to be repressed upon PEG treatment (Zhou et al., 2020). Meanwhile, the expressions of peroxisome-related genes, including *GmCSD1a/b*, *GmCSD2a/b/c*, and *GmCCS*, were upregulated upon PEG treatment (Zhou et al., 2020). Using GFP as the reporter in transient expression assays in Arabidopsis, *GmCSD1a/b*, *GmCSD2a/b/c*, and *GmCCS* were shown to be the cleavage targets of gma-MIR398c (Zhou et al., 2020). Composite soybean plants with transgenic roots overexpressing gma-MIR398c were more sensitive to PEG-simulated drought stress compared to the wild-type and *gma-MIR398c-*knockout mutants (Zhou et al., 2020). It was also suggested that certain *GmCSD1a/b* splicing variants may bypass the targeting by gma-MIR398c and thus bring forth another perspective on the regulation of drought stress responses (Zhou et al., 2020). In another global identification of miRNAs in soybean, gma-MIR1508a was found to be repressed by chilling (Xu et al., 2016). Later, by experimental validation, it was found that the expression of gma-MIR1508a was repressed by PEG treatment but induced by cold treatment (Sun et al., 2020). The overexpression of gma-MIR1508a in soybean reduced its tolerance to drought but improved its tolerance to cold stress (Sun et al., 2020). In the gma-MIR1508a over-expresser, the expressions of two *pentatricopeptide repeat* (*PPR*) genes (*Glyma.16G162100* and *Glyma.09G256600*) and four growth-related genes (*Glyma.17G065400*, *Glyma.08G028500*, *Glyma.07G001100*, and *Glyma.08G225900*) were repressed (Sun et al., 2020). The cleavage of *Glyma.16G162100* by gma-miR1508a was revealed by 5’rapid amplification of complementary DNA ends (5′RACE; Sun et al., 2020). However, how PPRs regulate stress tolerance in soybean remained unclear. Besides the global identification of stress-responsive miRNAs, there are also in-depth functional studies of miRNAs in soybean under stress. For example, miR172a was found to be induced in soybean seedlings treated with salt or drought stress, and involved in long-distance signaling (Pan et al., 2016). Composite soybean plants with transgenic hairy roots overexpressing *pre-miR172a* had enhanced tolerance to salt stress (Pan et al., 2016). *SSAC* (*salt suppressed AP2 domain-containing*) *1* (*Glyma.11G053800*) was predicted as the target of miR172a by degradome analysis (Pan et al., 2016). As a confirmation, in contrast to miR172a, the expression of *SSAC1* was repressed by salt stress while composite soybean plants with *SSAC1*-RNAi transgenic hairy roots had enhanced tolerance to salt stress (Pan et al., 2016). In addition, the transcript level of *SSAC1* was found to be reduced in both roots and leaves of composite soybean plants with transgenic hairy roots overexpressing *pre-miR172a* (Pan et al., 2016). Therefore, it was proposed that the root-expressed miR172a may be transported to the shoot to regulate the transcript level of *SSAC1* (Pan et al., 2016).

#### Biotic Stress

Besides abiotic stress, miRNAs are also regulators of biotic stress tolerance in soybean. Soybean cyst nematode (SCN) is a major soybean pest causing significant yield loss (Shaibu et al., 2020). The miR159 family was found to be responsive to cyst nematodes in genome-wide miRNA identification studies (Li et al., 2012; Tian et al., 2017). Among the members of the miR159 family, the mature miR159-3p was found to be abundant in plants (Millar et al., 2019). In a study of miR159-3p using transgenic soybean hairy roots, it was found that the overexpression of pre-miR159a could improve the resistance to cyst nematodes (Lei et al., 2021). Using psRNATarget, *GAMYB* genes were predicted to be the targets of miR159-3p (Lei et al., 2021). By 5’RACE, six *GAMYB* genes, *Glyma.04G125700*, *Glyma.06G312900*, *Glyma.13G073400*, *Glyma.13G187500*, *Glyma.15G225300*, and *Glyma.20G047600*, which encode *GmMYB33c*, *GmMYB33d*, *GmMYB33b*, *GmMYB33e*, *GmMYB33f*, *GmMYB33a*, respectively, were shown to be the cleavage targets of miR159-3p (Lei et al., 2021). As expected with the targeting of these transcripts by miR159-3p, the expression of miR159-3p was repressed during SCN infection while these target genes were upregulated (Lei et al., 2021). Compared to the wild-type, without SCN infection, these target genes were down-regulated in pre-miR159a/b/c/d/e/f over-expressers while up-regulated in the short tandem target mimic (STTM) lines of miR159a-3p/e-3p and miR159b-3p/f-3p (Lei et al., 2021). In addition, it was found that the abundance of miR159 was significantly induced by gibberellin (GA) at 10 μM, which also led to the down-regulation of the expressions of the *MYB* genes (Lei et al., 2021). GA treatment enhanced the resistance to SCN by soybean plants. This study shows the regulation of cyst nematode resistance through the interactions among a phytohormone, an miRNA and transcription factors, although the target genes of these MYB transcription factors are not clear. These interactions should also be taken into consideration when designing molecular breeding programs for desirable traits.

#### The Regulation of Growth

Besides regulating stress tolerance, the overexpression of gma-miR1508a also conferred dwarfism and increased cell wall thickness (Sun et al., 2020). It was proposed that the dwarf plants may have a higher transpiration rate and water loss under drought compared to the wild-type, thus resulting in drought sensitivity (Sun et al., 2020). Another example of growth-regulatory miRNA is Gma-miR156b which delays the flowering time of soybean but improves the yield (Cao et al., 2015; Sun et al., 2019). The overexpression of Gma-miR156b conferred enhanced stem thickness, branching, and yield compared to the wild-type, without affecting the height of the plant (Sun et al., 2019). The number of pods, number of seeds, seed length, seed width, seed thickness, seed weight, and yield were all increased in the over-expresser compared to the wild-type (Sun et al., 2019). Such a combination of traits is favorable for breeders. Gma-miR156b overexpression also enhanced the meristematic activity at the vegetative growth stage (Sun et al., 2019). By 5’RACE, 15 *GmSPL (Glycine max SQUAMOSA PROMOTER BINDING PROTEIN-LIKE*) genes were found to have their transcripts cleaved by Gma-miR156b (Sun et al., 2019). Among the GmSPL proteins, GmSPL9d was shown to interact with GmWUSa/b, which are the transcription factors defining the shoot stem cell niche and are involved in axillary meristem initiation (Sun et al., 2019).

#### The Regulation of Nutrient Uptake

Nodulation is an important strategy by soybean to acquire organic nitrogen and is a trait selected for during breeding (Kueneman et al., 1984). In soybean, miR482, miR1512, and miR1515 were induced by *Bradyrhizobium japonicum* inoculation in root (Li et al., 2010). Using transgenic soybean hairy roots, the nodule number was enhanced by either the overexpression of miR482 or miR1515 under a constitutive promoter or the expression of miR482 or miR1515 under a nodulation-inducible promoter (Li et al., 2010). By using 5’RACE, *Glyma.12G28730*, which encodes a GSK3 (glycogen synthase kinase 3)-like kinase, and *Glyma.09G02920*, which encodes a putative Dicer-like protein, were found to be the cleavage targets of miR482 and miR1515, respectively (Li et al., 2010). In soybean root hair, gma-miR2606b and gma-miR4416 were found to be repressed upon *B. japonicum* inoculation (Yan et al., 2016). However, the overexpression of gma-miR2606b and gma-miR4416 led to increased nodule number and decreased nodule number, respectively (Yan et al., 2016). This could be due to the fact that the overexpression of gma-miR2606b led to the repression of *Glyma.07G02290*, which encodes a mannosyl-oligosaccharide 1, 2-alpha-mannosidase (MNS), while the overexpression of gma-miR4416 led to the repression of *Glyma.11G29920* (*GmRIP1*) which encodes a putative rhizobium-induced peroxidase (RIP; Yan et al., 2016). These same gene targets of miR4416 and miR2606b were also predicted by the Parallel analysis of RNA Ends (PARE) library (Yan et al., 2016). As a result of the repression of gma-miR2606b upon *B. japonicum* inoculation, the expression of *GmRIP1* was induced in the root hair (Yan et al., 2016).

The miRNAs gma-miR171o and gma-miR171q were found to have differential expressions between uninoculated roots and nodules resulting from *B. japonicum* inoculation (Hossain et al., 2019). Gma-miR171o has reduced expression in nodules compared to uninoculated roots while the opposite trend was observed for gma-miR171q (Hossain et al., 2019). However, the overexpression of either gma-miR171o or gma-miR171q in transgenic soybean hairy roots could both inhibit nodulation upon *B. japonicum* inoculation (Hossain et al., 2019). PARE analysis revealed that *GmSCL6-1* (*Glycine max Scarecrow like 6–1*) and *GmNSP2* (*Glycine max Nodulation-signaling pathway 2*), which encode GRAS-family transcription factors, were the possible targets of gma-miR171o and gma-miR171q, respectively (Hossain et al., 2019). In transgenic soybean hairy roots, the overexpression of gma-miR171o and gma-miR171q led to the repression of *GmSCL6-1* and *GmNSP2.1*, respectively (Hossain et al., 2019). Using GFP as the cleavage reporter, transient expressions of gma-miR171o or gma-miR171q with *GmSCL6-1* or *GmNSP2.1*, respectively, in *Nicotiana benthamiana* leaves demonstrated the cleavage of *GmSCL6-1* transcripts by gma-miR171o and those of *GmNSP2.1* by gma-miR171q (Hossain et al., 2019). When the construct of *GmSCL6-1* or *GmNSP2.1* with a mutated cleavage site was transformed into soybean hairy roots, nodulation induced by *B. japonicum* inoculation was enhanced (Hossain et al., 2019). Altogether, these results indicated that miR171o and gma-miR171q negatively regulate nodulation by cleaving the transcripts of *GmSCL6-1* and *GmNSP2.1*, respectively. In another study, miR399b was found to be induced in soybean roots inoculated with *Sinorhizobium fredii*, with successful nodulation (Fan et al., 2021). Using transgenic soybean hairy roots, it was shown that the overexpression of miR399b improved the growth and nutrient acquisition of *S. fredii*-inoculated plants, as reflected in the enhanced whole-plant inorganic phosphate (Pi) concentration, whole plant ureide concentration, nodule number and leaf node number (Fan et al., 2021). The function of miR399b was further confirmed by the miR399b knock-down mutant in which opposite trends of the above parameters were observed (Fan et al., 2021). In uninoculated roots, inoculated roots and nodules, miR399b had opposite expression trends to those of *GmPHO2a* and *GmPHO2b*, which inhibit high-affinity Pi transporters (Fan et al., 2021). The cleavage of *GmPHO2a* and *GmPHO2b* transcripts by miR399b was validated by 5’RACE (Fan et al., 2021). It was then suggested that miR399b promotes Pi uptake by the plant by negatively regulating *GmPHO2a* and *GmPHO2b*. This enhanced Pi uptake probably in turn promoted nitrogen fixation in nodules.

The above examples show the capacity of a single miRNA to regulate multiple transcripts and multiple traits simultaneously, such as both Pi and nitrogen acquisitions, to improve the nutritional status of soybean. The interaction of soybean with rhizobia to form nitrogen-fixing nodules is an important trait for improving the nutritional status of the crop. Accumulating evidence shows that such an interaction is regulated by ncRNAs. It should be noted that usually one single ncRNA could regulate multiple transcripts, which could be useful for improving the efficiency of molecular breeding.

#### The Regulation of Soybean Seed Coat Color by siRNAs

Seed coat color is also an agronomic trait of interest to breeders (Heiser, 1988). *GmDCL2* (*Glycine max DICER-LIKE2*) encodes a dicer-like protein which mediates the generation of 22-nucloetide siRNAs from long inverted repeats-derived transcripts (Jia et al., 2020). When both copies of *GmDCL2* (*GmDCL2a* and *GmDCL2b*) were mutated by CRISPR/Cas9, the seed turned from yellow to brown (Jia et al., 2020). The levels of 22-nucleotide siRNAs in the *Gmdc2a*/*2b* mutant were greatly reduced (Jia et al., 2020). Specifically, a series of 22-nucleotide siRNAs from the antisense *CHS1* (*chalcone synthase 1*) region and the sense *CHS3* region were found in the wild-type but not in the *Gmdc2a*/*2b* mutant (Jia et al., 2020). Besides, other 21-nucleotide long secondary siRNAs from both antisense and sense strands of other *CHS* genes such as *CHS2*, *CHS7*, and *CHS8* were also absent in the *Gmdc2a*/*2b* mutant (Jia et al., 2020). It was reasoned that the disruption of siRNA generation led to the increase in *CHS* mRNA levels in the seed coat and resulted in the darker seed coat color (Jia et al., 2020). These observations suggest the negative role of siRNAs in the stability of their target mRNAs. This example also shows the power of transcriptional regulation of multiple genes by ncRNAs.

Examples of agronomic traits regulated by ncRNAs in soybean are listed in Table 1.

**Table 1:** Examples of soybean ncRNAs shown to regulate various agronomic traits.

Regulated agronomic trait	RNA type	Gene/locus	Description	References
Abiotic stress tolerance	miRNA	gma-miR1508a	Expression repressed by PEG but induced by cold treatment. Overexpression in soybean reduced tolerance to drought but improved tolerance to cold stress.	Xu et al., 2016; Sun et al., 2020.
		gma-miR398c	Expression repressed by PEG treatment. Overexpression in transgenic soybean hairy roots increased sensitivity to PEG-simulated drought stress.	Zhou et al., 2020
		miR172a	Expression induced by salt or drought stress in soybean seedlings. Composite soybean plants with transgenic hairy roots overexpressing pre-miR172a had enhanced tolerance to salt stress.	Pan et al., 2016
	lncRNA	lncRNA77580	Expression level was repressed in roots after NaCl treatment. Overexpression and large-fragment deletion of *lncRNA77580* had opposite effects on the expressions of several of the neighboring genes.	Niu et al., 2021
Biotic stress tolerance	miRNA	miR159a	Expression repressed by SCN infection. Overexpression of pre-miR159a could improve the resistance to cyst nematode.	Lei et al., 2021
Plant architecture		gma-miR1508a	Overexpression of gma-miR1508a also conferred dwarfism and thickened cell walls.	Sun et al., 2020
		gma-miR156b	Overexpression of gma-miR156b enhanced stem thickness, branching, and yield compared to the wild-type, while the height of the plant was not affected.	Sun et al., 2019
Nodulation		miR482	Overexpression in transgenic soybean hairy root or nodulation-induced expression enhanced nodule number. Targets *GSK* for cleavage.	Li et al., 2010
		miR1515	Overexpression in transgenic soybean hairy root or nodulation-induced expression enhanced nodule number. Targets *Glyma.09G02920*, which encodes a putative Dicer-like protein, for cleavage.	Li et al., 2010
		gma-miR2606b	Expression was repressed by *B. japonicum* inoculation in soybean root hair. Overexpression led to increased nodule number and repression of *Glyma.07G02290* which encodes MNS.	Yan et al., 2016
		gma-miR4416	Expression was repressed by *B. japonicum* inoculation in soybean root hair. Overexpression led to reduced nodule number and repression of *Glyma.11G29920* (*GmRIP1*).	Yan et al., 2016
		gma-miR171o	Reduced expression in nodules compared to uninoculated roots. Overexpression in transgenic soybean hairy roots inhibited nodulation upon *B. japonicum* inoculation. *GmSCL6-1* transcripts were the cleavage target.	Hossain et al., 2019
		gma-miR171q	Reduced expression in nodules compared to uninoculated roots. Overexpression in transgenic soybean hairy roots inhibited nodulation upon *B. japonicum* inoculation. *GmNSP2.1* transcripts were the cleavage target.	Hossain et al., 2019
Nodulation, phosphate uptake		miR399b	Overexpression of miR399b improved the growth and nutrient acquisition of *S. fredii*-inoculated plants. Mediated the cleavage of *GmPHO2a* and *GmPHO2b* transcripts.	Fan et al., 2021
Seed coat color	siRNA	siRNAs from sense and antisense strands of *CHS* genes	The mutation of *GmDCL2a* and *GmDCL2b* reduced the siRNA generation and led to the increase in *CHS* mRNAs.	Jia et al., 2020

### Interactions Between Regulators

LncRNAs and miRNAs are both post-transcriptional regulators. From the above examples, it could be observed that lncRNAs could interact with miRNAs which regulate the levels of protein-coding transcripts (Lu et al., 2020). Histone modification has been known as a mechanism of regulating plant growth, development, and the adaptations to abiotic and biotic stresses (Ashapkin et al., 2020; Luo et al., 2020; Ueda and Seki, 2020). The interplay between lncRNAs and miRNAs has been recently reviewed (Meng et al., 2021). Besides the competition between lncRNAs and miRNAs to bind their common target transcripts as discussed above, it has also been suggested that: 1) lncRNAs could be the targets of miRNAs to generate phasiRNAs; 2) lncRNAs could regulate pre-miRNA processing; 3) lncRNAs could act as target mimics of miRNAs; and 4) lncRNAs could inhibit miRNA expressions (Meng et al., 2021). Although the functional significance of such interactions has been largely unknown, the synergy brought forth by the interactions among ncRNAs should not be ignored when choosing the appropriate ncRNA targets for molecular breeding.

## Post-translational Regulation of Proteins

In order to cope with the changing environment, living organisms have evolved to respond instantaneously to external stimuli. It has been previously reviewed that plants could respond to stimuli in less than a second (Guo et al., 2015). Post-translational modifications of proteins provide a quick and flexible way to fine-tune the functions of proteins during their biosynthesis upon the sensing of an altered cellular microenvironment, and enable the plant to respond quickly, allowing for easy cultivation. Such quick modifications then mediate the transmission of signals for downstream responses. Post-translational regulations include chemical modifications, alteration in subcellular localizations, as well as regulation of the orientation or stability of proteins (Moore and Free, 1985).

Since the cell membrane is the front line in perceiving external stimuli, many cell membrane-associated proteins are known to be regulated post-translationally to achieve quick functional fine-tuning. Examples of these proteins will be discussed below.

### Signal Transduction—The Quick Functional Fine-Tuning by Altering the Nucleotide-Binding Property and Subcellular Localization of GTP-Binding Proteins

GTP-binding proteins (G-proteins) are the molecular switches in various signaling pathways. A well-known example of G-proteins is the membrane-associated trimeric G-protein, which consists of three subunits (α, β, and γ). When the α subunit binds with guanosine diphosphate (GDP), the β and γ subunits will associate with the complex to render the whole G-protein inactive to cease signaling. Upon perceiving an external stimulus, GDP will be phosphorylated to GTP and the GTP-bound α subunit would dissociate from the β and γ subunits. All three subunits are then active and can mediate responses to growth, hormonal controls, and stresses (Sharma, 2020). Arabidopsis G-protein α subunit (GPA1) regulates seed germination and root cell division, seed and fruit development, nitrate and phosphate responses, light responses and abscisic acid (ABA) signaling for stomatal opening (Ullah et al., 2002; Chen et al., 2006; Mishra et al., 2006; Chakraborty et al., 2015). Activated and dissociated G-protein β subunit (GPB1) was reported to regulate the development of leaf, flower and fruit, auxin and phototropism, and brassinosteroid (BR) signaling by interacting with BES1 which is the key transcription factor of BR signaling (Lease et al., 2001; Mudgil et al., 2009; Kansup et al., 2014; Zhang et al., 2018). The Arabidopsis trimeric G-protein γ subunit (AGG3) was reported to regulate stomatal closure in response to ABA (Chakravorty et al., 2011). In addition, the rice G-protein γ subunit (DEP1) was reported to regulate nitrogen sensing and thus affect grain yield (Sun et al., 2014).

In addition to trimeric G-proteins, unimolecular G-proteins associated with the plasma membrane through their regulators were also reported to participate in the adaptations to biotic and abiotic stresses. Rice YchF1 (OsYchF1) is an ancient unconventional G-protein conserved among the three domains of life (Cheung et al., 2016). It possesses a unique G4 motif which allows the protein to bind with both GTP and ATP (Cheung et al., 2016). OsYchF1 was first identified by its interaction with a rice GTPase (OsGAP1), which is a transcription activator that enhanced the resistance against *Xanthomonas oryzae* pv. *oryzae* (*Xoo*) in rice and *Pseudomonas syringae* pv. *tomato* (*Pst*) DC3000 in Arabidopsis (Cheung et al., 2010). Meanwhile, the ectopic expression of *OsYchF1* in Arabidopsis was reported to enhance the plant’s susceptibility to *Pst* DC3000 (Cheung et al., 2013). Furthermore, OsGAP1 was shown to be a positive regulator in salt stress response by inhibiting OsYchF1, which promoted salt sensitivity (Cheung et al., 2013; Yung et al., 2015). Upon wounding, OsGAP1 competed for the nucleic acid binding site at the TGS domain of OsYchF1, preventing GDP from binding to OsYchF1, and altering the subcellular localization of OsYchF1 from the cytoplasm to the plasma membrane (Cheung et al., 2010, 2016). These regulations targeted the GTPase activity of OsYchF1 (Cheung et al., 2010, 2016). Such a functional regulation by altering the nucleotide-binding property of the protein and its subcellular localization is an example of quick regulatory reactions in response to environmental stimuli.

### Signal Transduction—The Regulation of Hormone Signaling by Membrane-Bound Receptor Kinases

Membrane-bound kinases, including receptor-like kinases (RLKs), SNF1-related protein kinases (SnRKs) and histidine kinases (HKs), play crucial roles in abiotic stress responses (Chakradhar et al., 2019). RLKs harbor a conserved serine/threonine catalytic domain which transduces signals *via* phosphorylation and dephosphorylation in response to stimuli. RLKs have been reported to regulate various functions such as stomatal opening and avirulent (Avr) protein recognition (Chakradhar et al., 2019). For example, the hydrogen peroxide resistant 1 (GHR1) protein in the guard cell is involved in the ABA-dependent hydrogen peroxide-mediated pathway controlling stomatal opening (Hua et al., 2012). Another example is RLK7, which is involved in seed germination and confers oxidative stress tolerance in Arabidopsis (Pitorre et al., 2010).

SnRKs also participate in ABA signaling. The two groups of plant-specific proteins, SnRK1 and SnRK2, are activated by ABA through their interactions with protein phosphatase type 2C (PP2C). PP2C inhibits SnRK2 proteins by dephosphorylation. When the ABA level increases, cytoplasmic PYR/PYL/RCAR, which are ABA receptors, inhibit the activity of PP2C and in turn activate SnRK2 (Umezawa et al., 2009). The over-expression of *SRK2C* (*SNF1-related protein kinase 2C*), which encodes an SnRK2 protein in *Arabidopsis thaliana*, resulted in enhanced drought tolerance (Umezawa et al., 2004).

While RLKs and SnRKs are usually involved in ABA signaling, HKs are mostly reported as osmosensors and receptors for ethylene and cytokinin signaling. In Arabidopsis, ETR1 (ETHYLENE RESPONSE1) was reported to be an ethylene response regulator through its histidine kinase activity (Hall et al., 2012). The mutated etr1, with abolished kinase activity, had reduced responsiveness to ethylene, which is associated with the growth and stress responses of plants (Hall et al., 2012). while AHK2 (Arabidopsis histidine kinase 2), AHK3 and AHK4 were reported to be cytokinin receptors (Nongpiur et al., 2012), and can trigger downstream signaling in response to various abiotic stresses including cold, drought and salt (Chakradhar et al., 2019). *Athk2* and *Athk3* mutants exhibited drought and salt tolerance phenotypes (Nongpiur et al., 2012). In addition, the over-expression of *AtHK1* in Arabidopsis resulted in enhanced drought tolerance (Tran et al., 2007). In *Oryza sativa* and *A. thaliana*, the two-component system of histidine-aspartate relay signaling was reported (Pareek et al., 2006). HKs normally auto-phosphorylate the conserved histidine residue in the kinase domain. Then, upon stimulus perception, the phosphate group on the histidine residue is transferred to the conserved aspartate residue within the receiver domain (Pareek et al., 2006).

Examples of membrane-bound kinases that regulate the stress responses of plants as a part of hormone signaling pathways, and their homologous proteins in soybean, are summarized in Table 2.

**Table 2:** Examples of proteins which are subjected to post-translational modifications and their homologs in soybean.

Protein type	Functions	Characterized proteins	Soybean homologs^#^	References
Membrane-bound receptor kinases	Involved in ABA-dependent hydrogen peroxide-mediated pathway controlling stomatal opening	Arabidopsis guard cell hydrogen peroxide resistant 1 (GHR1) (accession number: AT4G20940)	Glyma.09G024900.2.p	Hua et al., 2012
	In the presence of ABA, SRK2C (SnRK2 family member) would be released from the inhibition by PP2C The over-expression of *SRK2C* in transgenic *Arabidopsis* enhanced drought tolerance	Arabidopsis SRK2C (accession number: AT1G72910)	Glyma.02G208500.1.p	Umezawa et al., 2004, 2009
	Involved in ethylene signaling	Arabidopsis ETR1 (accession number: AT1G66340)	Glyma.12G241700.4.p	Hall et al., 2012
	Cytokinin receptors; *Athk2* and *Athk3* mutants exhibit drought and salt-tolerant phenotype and thus are negative regulator in salinity and osmotic stresses	Arabidopsis AHK2 (accession number: AT5G35750); Arabidopsis AHK3 (accession number: AT1G27320)	GmHK2: Glyma.14G007100.2.p; GmHK3: Glyma.05G148100.1.p	Nongpiur et al., 2012
MATE transporters	Transports citrate out of the plant and thus enhances aluminum tolerance	Rice bean (*Vigna umbellata)* VuMATE1 (accession number: KM090855) and VuMATE2 (accession number: KR494281)	VuMATE1 homolog: Glyma.12G237400.2.p VuMATE2 homolog: Glyma.02G181800.1.p	Liu et al., 2013, 2018
	Mediates iron homeostasis	Arabidopsis FRD3/ AtDTX43 (accession number: AF448231)	Glyma.09G102800.3.p	Rogers and Guerinot, 2002
	Enhances drought, salt and cold stress tolerance by translocating ABA	Cotton (*Gossypium hirsutum) Gh_D06G0281* (*DTX/MATE*) gene (accession number: LOC121218223)	Glyma.04G097900.1.p	Lu et al., 2019
	Mediates salicylic acid (SA)-dependent signaling for disease resistance	Arabidopsis MATE EDS5/ AtDTX47 (accession number: AF416569)	Glyma.11G112200.1.p	Nawrath et al., 2002

# The soybean homologs were identified by using the protein BLAST tool provided by the National Center for Biotechnology Information (U.S. National Library of Medicine, https://blast.ncbi.nlm.nih.gov/Blast.cgi?PAGE=Proteins) and Phytozome 13 (https://phytozome-next.jgi.doe.gov/).

### Substrate Transport—The Regulation of the Orientation of Transport by Multidrug and Toxic Compound Extrusion Transporters Through Protonation

MATE transporters are members of one of the major transporter families found in the three domains of life, having conserved protein structures from Archaea to Eukaryotes. In plants, MATEs have been reported to transport various substrates including phytohormones, antibiotics, ion chelators, alkaloids and flavonoids, and are involved in leaf senescence, iron homeostasis, aluminum tolerance, and even synthesis of phytohormones (Takanashi et al., 2014; Kusakizako et al., 2020; Ng et al., 2021). They have been shown to play roles in both abiotic stresses and biotic stresses in plants (Table 2). For example, in *Vigna umbellata*, VuMATE1 and VuMATE2 were found to export citrate out of the plant to chelate aluminum ions and thus conferred aluminum tolerance (Liu et al., 2013, 2018). Besides aluminum, MATE transporters were also reported to mediate iron homeostasis in plants (Rogers and Guerinot, 2002). The cotton MATE protein, Gh_D06G0281, was shown to mediate the transport of ABA and enhance the tolerance to drought, salt, and cold stresses in transgenic *Arabidopsis* (Lu et al., 2019). Besides ABA, MATE transporters have also been demonstrated to be involved in salicylic acid (SA) signaling. In *Arabidopsis*, EDS5 (ENHANCED DISEASE SUSCEPTIBILITY 5) was found to be a MATE homolog that mediates SA-dependent signaling for disease resistance (Nawrath et al., 2002). These stress-related MATE proteins and their soybean homologs are listed in Table 2.

In terms of the protein structure, MATE proteins generally comprise 12 transmembrane domains (Piddock, 2006). The transport of substrates is driven by the electrochemical gradient across the biological membrane where the MATE transporter is localized (Meyer et al., 2009). Based on previous studies, it has been revealed that MATE transporters in eukaryotes generally transport the substrates in exchange for H^+^ while those in prokaryotes could transport their substrates in exchange for H^+^ or Na^+^ (Omote et al., 2006). Based on the crystal structure, it was reported that the 12 conserved transmembrane domains (TMs) are arranged in a unique topology with the N-lobe consisting of TM1 to TM6 and the C-lobe consisting of TM7 to TM12 (Jagessar et al., 2019). The N- and C-lobes are linked by a cytoplasmic loop and display an intramolecular twofold symmetry (Jagessar et al., 2019). Such an orientation of the TMs forms a large central cavity for substrate transportation (Jagessar et al., 2019). This outward-facing conformation was first reported with the crystallized protein in free form, without a bound substrate. Through a double electron–electron resonance (DEER) study, the inward-facing conformation was found to be favored at pH 4, while the outward-facing conformation was found to be favored at pH 7.5 with substrate binding. Altogether, it implies that the protonation state of the different domains of the MATE protein regulates its orientation within the membrane where it is localized (Jagessar et al., 2019). Besides the protonation state, the hydration of the protein and the lipid composition of the membrane also influence the functions of MATE transporters. The hydration of the N-lobe cavity of a MATE protein could weaken substrate binding and even lead to the release of the substrate before lipid intrusion (Nishima et al., 2016). As different lipid species bear different pKa values of their headgroups, variations in the lipid composition of the membrane under different conditions would thus affect the substrate transport efficiency (Nishima et al., 2016). These properties of MATE transporters reveal their capacity to respond quickly to the changing cellular environment and thus their potential to adapt to different cultivation conditions.

Examples of MATE transporters that regulate the stress responses of plants and their homologous proteins in soybean are summarized in Table 2.

## Translational regulators Are Stress-Responsive

The power of translational regulators lies in their capacity to regulate the protein abundance from multiple genes. The alterations in ribosomal proteins, translation elongation factors, translation initiation factors, and ribosomes and ribosomal RNAs could influence translation efficiency, which could be part of stress-coping mechanisms.

### Ribosomal Proteins

#### Abiotic Stress

Ribosomal proteins have been reported to regulate abiotic stress responses in soybean, such as cold stress responses. Several cold-sensitive yeast mutants have been shown to be defective in the assembly of ribosomal subunits (Bayliss and Ingraham, 1974). A ribosomal protein L34-like protein in soybean, GmSOL34, was induced in root tips and embryonic axes by low temperatures. The expression level of *GmSOL34* was positively correlated to the increased duration of imbibition (Cheng et al., 2010). By overexpressing the sense and antisense in *Arabidopsis*, the transgenic *SOL34ox* plants showed a negative correlation between the increased *SOL34* level and the survival rate of *Arabidopsis* under low-temperature conditions, while the transgenic *Arabidopsis* overexpressing the antisense *SOL34* exhibited a better adaptation to freezing temperatures than the wild type plants during short cold imbibition (Cheng et al., 2010). Therefore, it was suggested that *SOL34* might play a negative role in plant response to temperature stress.

Similarly, the soybean ribosomal proteins *GmRPS13*, *GmRPS6*, and *GmRPS37* were found to be involved in cold stress adaptation (Kim et al., 2004). Unlike cold-stress proteins such as the soybean cold-stress response protein SRC1, the genes encoding these ribosomal proteins were not induced at the early stage of the cold stress treatment until three days after treatment started. RPS6 is in the mRNA-binding site of the 40S subunit of the cytosolic ribosome (Nygård and Nilsson, 1990). It is proposed that the late induction of *GmRPS13*, *GmRPS6*, and *GmRPS37* might assist in the proper ribosome assembly and protein translation under cold stress (Kim et al., 2004).

#### Biotic Stress

Components in the translational machinery provide a structure for ribosomes or their associated factors and play a crucial role in translation. As mentioned above, the translational machinery might play a role in stress-coping, as some translation-related genes are stress-responsive. For example in soybean, the ribosomal protein L2 showed a transient down-regulated mRNA and protein levels upon encountering the pathogen *Phytophthora sojae* and its elicitors, as well as exposure to heavy metals (Ludwig and Tenhaken, 2001). As demonstrated using the yeast ribosome model, ribosomal protein L2 is essential for the peptidyl-transferase activity of ribosomes (Diedrich et al., 2000). By measuring the autoradiography of the newly synthesized proteins incorporating ^35^S-labeled amino acids, it is remarkable that the *rpL2* mRNA down-regulation was well-correlated with the transient loss of newly synthesized proteins. This observation persisted with the programmed cell death-induced expression of *DD1-51* (EMBL: AJ289152), suggesting the presence of a block on protein synthesis at the translation stage but not on the transcription of mRNAs after the *Phytophthora sojae* infection (Ludwig and Tenhaken, 2000). It was hypothesized that the long lag time between mRNA accumulation and the appearance of newly synthesized proteins was caused by the down-regulated rpL2. Such down-regulation of the ribosomal protein L2 might directly lead to less efficient translation of cellular mRNAs, which favors the re-modeling of stress-coping mechanisms in cells (Ludwig and Tenhaken, 2001).

### Translation Elongation Factors

#### Abiotic Stress

Besides ribosomal proteins, translation elongation factors are also reported to be involved in stress adaptation. For example, the Arabidopsis *los1* mutant, which is deficient in producing eukaryotic translation elongation factor 2 (eEF2), was shown to have impaired cold sensing (Guo et al., 2002). It was further reported that the cold-induced expression of the early response transcriptional activators, C-repeat/dehydration responsive element-binding factor1 (CBF/DREB1), was enhanced by the *los1-1* mutation. Protein synthesis is normal in *los1–1* mutant plant at warm temperatures but is blocked in the cold as shown with the autoradiography of newly synthesized proteins incorporating ^35^S-labeled amino acids. The altered translation efficiency might be involved in stress adaptation in the plant (Guo et al., 2002).

The comparison of proteomes among different salt-tolerant genotypes of soybean revealed a possible involvement of the translational machinery in salt stress adaptability (Ma et al., 2012). Multiple translation-related factors involved in the initiation and elongation of the peptide chain showed altered abundance when under salt stress (Ma et al., 2012). A similar inhibition of translation elongation by eEF2 under cold stress was observed in the human cells when compared to that in the Arabidopsis *los1-1* mutant (Guo et al., 2002; Knight et al., 2015). It was reported that the phosphorylation of eEF2 by the eEF2 kinase reduced the physical interference between mRNA-bound ribosomes around the start codon and thus slowed down the translation activity by inhibiting ribosome translocation under cold stress. Besides eEF2, elongation factor 1α (EF1α) was also reported to have unstable expression under stress conditions (Chung et al., 2009; Saraiva et al., 2014). For example, *SLTI100*, which encodes EF1α in soybean, was reported to be induced under stresses including low temperature, salinity, drought, and ABA treatment (Chung et al., 2009). It was reported that the mRNA levels of the soybean EF1α family were responsive to drought stress or ABA treatment, by being either up- or down-regulated (Gao et al., 2019). Under salt stress, the genes were shown to be up-regulated (Gao et al., 2019). Among the genes of the soybean EF1α family, *GmEF4* was found to be induced under salt stress (Gao et al., 2019). Composite soybean plants having *GmEF4*-overexpressing hairy roots survived better under drought and high salinity compared to the empty vector control as revealed by the biomass, proline contents, and H_2_O_2_ and O^−^ contents (Gao et al., 2019). Other than its involvement in abiotic stress, GmEF1A is also the host factor of the soybean mosaic virus (SMV) during viral pathogenesis (Luan et al., 2016).

#### Biotic Stress

Translation elongation factors have also been demonstrated to regulate the responses to biotic stress in soybean. It was shown that the virus-induced gene silencing (VIGS) of *GmEF1A* in soybean did not alter the morphology of the soybean plant but alleviated soybean mosaic virus (SMV) accumulation, ER stress accumulation, and the SMV-induced cell death, probably the result of interrupting the interaction between the potyviral P3 protein and GmEF1A (Luan et al., 2016). Similarly, knocking down *GmEF1B*, which encodes a guanine nucleotide exchange factor that restores the GTP moiety to reactivate EF1A, could also enhance the soybean resistance to SMV (Luan et al., 2016).

### Translation Initiation Factors

#### Abiotic Stress

Besides translation elongation, translation initiation factors could also regulate stress responses in soybean (Alam et al., 2010; Yin and Komatsu, 2015; Gallino et al., 2018; Cho et al., 2019; Wang et al., 2021). eIF4G was reported to act as a hub in translation initiation and mediate the recruitment of additional initiation factors (Sonenberg et al., 1978). The eukaryotic translation initiation factor iso4G (GmeIFiso4G-1a) exhibited a specific drought induction profile in the slow-wilting soybean cultivar N7001 compared to the drought-sensitive TJS2049 soybean cultivar (Gallino et al., 2018). The heterologous expression of soybean *GmeIFiso4G-1a* in *Arabidopsis* improved the tolerance to osmotic, salt, drought, and low-temperature stresses (Gallino et al., 2018). In another report, it was shown that the *Arabidopsis* mutant *eifiso4G1*, a homolog of the soybean *GmeIFiso4G-1a*, had increased sensitivity to submergence due to the disruption of the interaction between eIFiso4G1 and Snf1-related protein kinase 1 (SnRK1), which phosphorylates eIFiso4G1 for translational regulation (Cho et al., 2019).

Flooding affected the mRNA levels of some genes that encode ribosomal proteins, and altered the abundances of nuclear proteins and phosphoproteins in the soybean root tip (Yin and Komatsu, 2015). For instance, under flooding, the mRNA levels of the ribosomal protein S24/S35 family and eukaryotic translation initiation factor 4G (eIF4G) were lowered although the protein levels were not affected (Yin and Komatsu, 2015).

Besides stress tolerance, translation elongation factors also regulate the growth and development of plants. For example, the knockout mutation of *Arabidopsis eIFiso4G*, which is an isoform of *elF4G*, reduced the plant’s adaptability to dehydration, as well as causing slower growth and development (Lellis et al., 2010). In another report, using proteomic analyses of the soybean root, it was shown that the level of eukaryotic translation initiation factor 5A (eIF5A) was decreased after flooding (Alam et al., 2010; Wang et al., 2021). Salt and heavy metal stress induced the mRNA levels of *OseIF5A-1* and *OseIF5A-2* in rice (Chou et al., 2004). In *Tamarix androssowii*, the overexpression of *TaeIF5A1* also enhanced the tolerance of the transgenic poplar plants to various abiotic stresses, including salt stress and cadmium stress (Wang et al., 2012).

#### Biotic Stress

Transgenic Arabidopsis with constitutively suppressed *AteIF5A-2* showed a reduction in pathogen growth and therefore stronger resistance against *Pseudomonas syringae* pv *tomato* (*Pst*DC3000), while the overexpression of *AteIF5A-2* showed similar susceptibility to the *Pst*DC3000 as the wild type (Hopkins et al., 2008). It was suggested that the eIF5A level might determine the rate of translation of the mRNA species required for specific stress responses. This example hints at the importance of the proper abundance of the translational regulators. Compared to the model plant *Arabidopsis*, the involvement of soybean translation initiation factors in regulating the responses to biotic stress is not well-studied. However, the example in *Arabidopsis* may indicate the direction for similar research in soybean.

### Ribosomes and Ribosomal RNAs

The abundance of the ribosomes and ribosomal RNAs could also be related to plant stress adaptations. There was an overall increase in the level of the ribosomal protein mRNAs and ribosomal RNAs (rRNAs) in soybean hypocotyls under synthetic auxin (2,4-dichlorophenoxy acetic acid) treatment, with a decreased proportion of the total RNA being poly(A)-tailed RNAs (Gantt and Key, 1985). A similar alteration in the rate of rRNA biogenesis was observed in rice under chilling stress at the pre-rRNA processing stage (Hang et al., 2018). It was proposed that the higher activity of RNA polymerase I and the higher expression levels of the ribosomal protein mRNAs led to the increased abundance of ribosomes, which in turn might be associated with the higher growth rate of the plant (Gantt and Key, 1985). On the other hand, when experiencing cold stress, the ribosome biogenesis rate might be reduced for better resource allocation to promote acclimation and survival at lower temperatures (Hang et al., 2018).

Examples of these translation regulators and their responses to various stresses are listed in Table 3.

**Table 3:** Examples of translation regulators in soybean.

Component in translation machinery	Protein name	Predicted mechanism	Stress	Responses upon stress	References
Ribosomal proteins	GmRPL2	Blocks translation despite stress-coping mRNA accumulation	*Phytophthora sojae* infection; heavy metal stress	Downregulation upon *Phytophthora sojae* infection and heavy metal stress	Ludwig and Tenhaken, 2001
	GmSOL34 (RPL34-like protein)	/	Cold stress	Upregulated at root tip and embryonic axes; overexpression of antisense *GmSOL34* in Arabidopsis reported with better adaptation to freezing temperature	Cheng et al., 2010
	GmRPS13 GmRPS6 GmRPS37	Might assist proper ribosome assembly and translation under cold stress	Cold stress	Upregulated after three days of treatment	Kim et al., 2004
Translation elongation factors	*Atlos1-1* (Homolog of Glyma.08G170000)	Required for proper protein synthesis under cold condition	Cold stress	Mutants with impaired cold sensing and signal transduction	Guo et al., 2002
	SLTI100 (GmEF1α)	/	Low temperature, salt, ABA, and drought stress	Upregulated under various abiotic stresses	Gao et al., 2019
	GmEF4 (GmEF1α family)	/	Drought and salt treatments	Overexpression in hairy root system reported with better survivorship under drought and high salinity	Gao et al., 2019
	GmEF1α	Silencing of GmEF1α diminishes its interaction with the potyviral P3 protein and leads to better soybean mosaic virus (SMV) resistance	Soybean mosaic virus (SMV) infection	Silencing of GmEF1α resulted in enhanced resistance to SMV	Luan et al., 2016
Translation initiation factors	GmeIFiso4G-1a	/	Osmotic, salt, drought, and low-temperature stresses	Overexpression in Arabidopsis reported with better survivorship under various abiotic stresses	Gallino et al., 2018
	AteIFiso4G1 (Homolog of GmeIFiso4G-1a)	Disruption of the interaction between eIFiso4G1 and Snf1-related protein kinase 1 (SnRK1) leads to altered translation dynamics	Flooding	Mutants more sensitive to submergence	Cho et al., 2019
	GmeIF4G	/	Flooding	Lowered abundance in mRNA level but not the protein level under flooding stress	Yin and Komatsu, 2015
	AteIFiso4G (Homolog of GmeIF4G)	/	Dehydration	Knockout mutant with reduced adaptability under dehydration stress	Lellis et al., 2010
	GmeIF5A	/	Flooding	Downregulated under flooding stress	Alam et al., 2010; Wang et al., 2021
	OseIF5A (Homolog of GmeIF5A)	/	Salt and heavy metal stress	Upregulated under salt and heavy metal stress	Chou et al., 2004
	TaeIF5A (Homolog of GmeIF5A)	TaeIF5A1 expression controlled by transcription factors, TaWRKYs and TaRAVs, in the ABA signaling pathway	Salt, PEG6000, NaHCO_3_, CdCl_2_, and ABA treatment	Overexpression enhances abiotic stress tolerance	Wang et al., 2012.
	AteIF5A-2 (Homolog of GmeIF5A)	/	*Pst*DC3000 infection	Downregulation enhances tolerance to *Pst*DC3000	Hopkins et al., 2008
Ribosomes and rRNAs	Ribosomes and rRNAs in soybean	Increased abundance of ribosomes might correlate with growth rate	Synthetic auxin treatment	Upregulated RPs and rRNAs under auxin treatment	Gantt and Key, 1985
	rRNAs in rice	Reduced ribosome biogenesis rate for re-directing resources to cope with stress	Chilling stress	Altered rRNA biogenesis rate	Hang et al., 2018

### Wild Soybean Germplasms as Important Genetic Resources

The above examples are largely from cultivated soybean (*Glycine max* [L.] Merr.) which have been more extensively studied compared to wild soybean (*Glycine soja* Sieb. and Zucc.). However, the genetic diversity of wild soybean germplasms (Lam et al., 2010; Xie et al., 2019) provides soybean molecular breeding with more possibilities. Although pan genome analyses did not show a significant differentiation of ncRNA compositions between the genomes of cultivated soybean germplasms and wild soybean germplasms (Liu et al., 2020b), novel ncRNA identifications had been reported in wild soybean (Chen et al., 2009; Zeng et al., 2012). By miRNA identification and degradome sequencing, known miRNAs as well as novel miRNAs were identified from the root of wild soybean seedlings (Zeng et al., 2012). Some of the novel miRNAs were found to be responsive to aluminum (Al) stress (Zeng et al., 2012). Similar to the observation in cultivated soybean, many of the miRNAs in wild soybean were predicted to target multiple transcripts (Zeng et al., 2012). In other studies, germplasm-specific expression patterns of ncRNA genes were also reported. For example, the lncRNA Gmax_MSTRG.19570 was induced in wild soybean W05 but not in cultivated soybean C08 upon salt stress (Lin et al., 2020). Similarly, several miRNAs, including miR156b, miR156f, miR160a, miR166i, miR390a, miR390e, miR390f, miR390g, miR394a, miR4413a, miR4416c, and miR5225, were found to have divergent expression trends between cultivated soybean C08 and wild soybean W05 upon salt stress (Li et al., 2022). These examples suggest the possibility to discover novel ncRNAs from wild soybean germplasms. In addition, the stress responses of the ncRNAs in wild soybean may be different from those in cultivated soybean. Hence, wild soybean germplasms shall provide ncRNA-based molecular breeding with more possibilities.

## Discussion

### Agronomic Traits and Their Post-transcriptional Regulators

Various agronomic traits of soybean including nodulation, nutrient uptake, stress tolerance, and seed color are regulated post-transcriptionally. Pilot studies showed that lncRNAs are responsive to various abiotic stresses including drought, salt, alkaline, and CO_2_ concentration (Lin et al., 2020; Niu et al., 2021). However, the regulation of biotic stress in soybean by lncRNAs is relatively unclear. Nevertheless, the functional study of lncRNA77580 in soybean revealed its regulation on neighboring genes (Niu et al., 2021). Such regulation gives hints to the mechanistic studies of stress-related lncRNAs. Compared to lncRNAs, the functions of miRNAs are more well-studied. Besides the regulation of abiotic and abiotic stresses (Li et al., 2012; Pan et al., 2016; Xu et al., 2016; Tian et al., 2017; Sun et al., 2020; Wu et al., 2020), the roles of miRNAs in regulating soybean nodulation and nutrient acquisition have also been reported (Li et al., 2010; Yan et al., 2016; Hossain et al., 2019; Fan et al., 2021). Plant species in the asterids clade are rich in siRNA22 loci in their genomes (Lunardon et al., 2020). Although soybean is not in the asterids clade, it also has abundant siRNA22 loci in the genome (Lunardon et al., 2020). In soybean, siRNAs were reported to regulate the seed coat color, which is a domestication-related trait (Heiser, 1988; Jia et al., 2020).

Upon stresses, protein modifications such as phosphorylation, nucleotide binding property, and the subsequent alteration of subcellular localization enable quick functional fine-tunes (Umezawa et al., 2004; Cheung et al., 2010, 2013, 2016; Yung et al., 2015; Chakradhar et al., 2019). A number of phytohormone signaling pathways, such as those of auxin, BR, ABA, ethylene, and cytokinin signaling, involve phosphorylation of the signaling components (Lease et al., 2001; Mudgil et al., 2009; Umezawa et al., 2009; Hall et al., 2012; Kansup et al., 2014; Zhang et al., 2018).

The regulations of the translational machinery in terms of ribosome abundance, ribosome assembly, and translation efficiency (Ludwig and Tenhaken, 2001; Guo et al., 2002; Kim et al., 2004; Hopkins et al., 2008; Hang et al., 2018) play important roles in balancing energy distributions upon stresses. The power of such regulations lies on the capacity to regulate protein abundance globally. Compared to the model plant Arabidopsis, the regulations of the translational machinery in soybean are less comprehensive. More mechanistic studies of such regulations in soybean shall provide important information for understanding the stress tolerance mechanism and selecting elite genes for molecular breeding.

**Figure 1:**
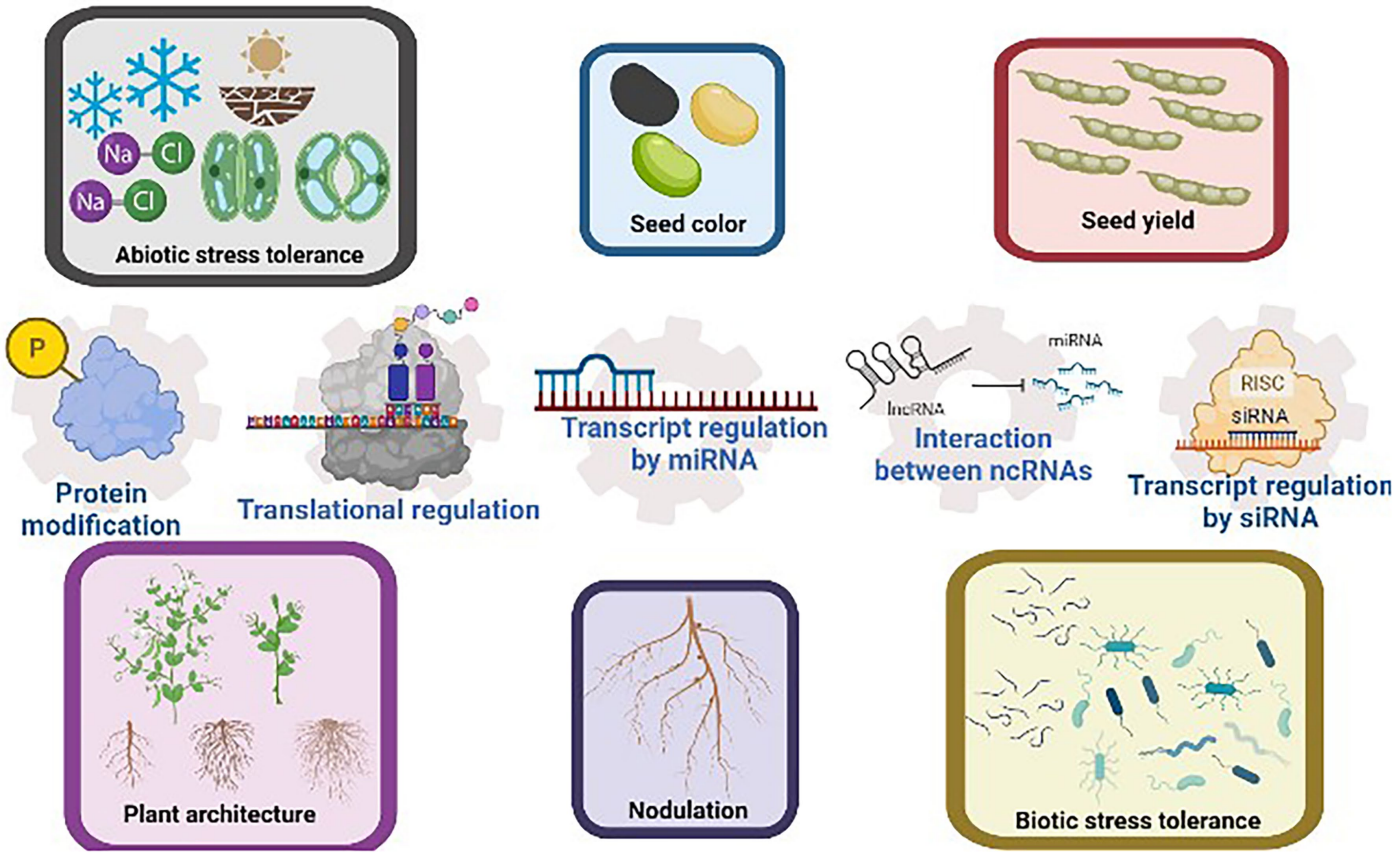
Common agronomic traits selected in soybean breeding include seed color and yield, plant architecture, nodulation efficiency, and the tolerance to abiotic and biotic stresses. These traits have been reported to be regulated by post-transcriptional regulatory mechanisms such as transcript regulation by ncRNAs, proteins modification, and translational regulation. The interaction between ncRNAs further increases the versatility of post-transcriptional regulations. This figure is created with BioRender.com.

Agronomic traits regulated by various post-transcriptional regulatory mechanisms are summarized in Figure 1.

### The Power of Post-translational Regulation-Related Genes in Facilitating Soybean Molecular Breeding

The goal of breeding usually is to stack multiple desirable traits in an individual cultivar. When a single trait is controlled by multiple genes, it poses a hurdle to breeding since gene stacking would be difficult. However, the above examples show that, usually, a single ncRNA could regulate the transcripts of multiple genes. Besides, regulators of the translation machinery can alter the translation efficiency of a set of proteins to allow better resource allocation and acclimation when under stress. Thus, selecting for genes related to post-translational regulations may effectively reduce the need for gene stacking. In addition to the capacity to regulate multiple genes and proteins, post-translational regulations also present other advantages. For example, ncRNAs could also be mobile to allow long-distance signaling. Such a property enables a more flexible choice of tissues for expressing the gene of interest. The ability of post-translational regulations to quickly fine-tune protein functions when under stress may also be a useful feature for developing breeding programs to produce stress-tolerant soybean accessions.

## Conclusion

Soybean is rich in nutrients including protein, oil, and health-beneficial secondary metabolites. However, the tolerance of soybean to stresses such as drought, salinity, and cyst nematode is of concern. Soybean breeding programs usually aim to produce soybean accessions with good nutritional properties and stress tolerance. With the increasing knowledge on soybean genomics, the selection of elite soybean genes for molecular breeding has been made more feasible. However, the need to stack multiple genes to achieve multiple desired traits in the same cultivar may pose difficulties for breeding. To reduce the need of stacking genes, the selection of genes related to post-transcriptional regulations may be advantageous. Many ncRNAs have been shown to regulate the transcripts of multiple genes. The regulation of the translation machinery also allows for the regulation of a set of proteins at a time. Besides, small ncRNAs act as long-distance signals and increase the flexibility in the choice of tissue for their expressions. The ability of post-translational modifications to quickly fine-tune protein functions when under stress also provides a choice of genes for improving the stress response capacity of soybean plants. These features make the genes related to post-transcriptional regulations highly suitable as targets for soybean molecular breeding.

## Author Contributions

H-ML planned and coordinated the writing. Y-SK put together the first complete draft. All authors contributed to the search of literature and writings. All authors contributed to the article and approved the submitted version.

## Funding

This work was supported by grants from the Hong Kong Research Grants Council Area of Excellence Scheme (AoE/M-403/16) and Lo Kwee-Seong Biomedical Research Fund to H-ML.

## Conflict of Interest

The authors declare that the research was conducted in the absence of any commercial or financial relationships that could be construed as a potential conflict of interest.

## Publisher’s Note

All claims expressed in this article are solely those of the authors and do not necessarily represent those of their affiliated organizations, or those of the publisher, the editors and the reviewers. Any product that may be evaluated in this article, or claim that may be made by its manufacturer, is not guaranteed or endorsed by the publisher.
